# Artificial intelligence and psychotherapy interventions

**DOI:** 10.1192/j.eurpsy.2025.221

**Published:** 2025-08-26

**Authors:** R. Karaliuniene

**Affiliations:** Clinic for Psychiatry and Psychotherapy, Rhein Mosel Clinic, Andernach, Germany

## Abstract

**Abstract:**

This presentation explores the intersection of psychotherapy interventions and AI, focusing on how AI can be leveraged to augment traditional therapeutic methods. AI-powered tools such as chatbots, virtual therapists, and predictive analytics will be covered. Additionally, the talk will address the ethical implications, challenges, and opportunities posed by the use of AI in mental health care, emphasizing the importance of maintaining the human element in therapy. Drawing on current research and case studies, possible opportunities of AI assisted therapists in identifying patterns in patient behavior, improving treatment efficacy, and reducing barriers to mental health care, especially in underserved populations will be explored. The presentation will conclude with a forward-looking perspective on how AI and psychotherapy can collaboratively evolve to shape the future of mental health care.

**Keywords:**

Artificial Intelligence, Psychotherapy, Mental Health, Chatbots, Virtual Therapy, Ethical Considerations, Personalized Treatment.

**Disclosure of Interest:**

None Declared

